# Safety and Nutritional Profile of Traditional Turkish Cheeses: A Comprehensive Study on Their Mineral Content, Heavy Metal Contamination, and Health Risks of Aho, Golot, and Telli

**DOI:** 10.1002/fsn3.70560

**Published:** 2025-07-20

**Authors:** Seyit Erol, Bayram Ürkek

**Affiliations:** ^1^ Engineering and Natural Sciences, Department of Food Engineering Gümüşhane University Gümüşhane Turkey; ^2^ Siran Mustafa Beyaz Vocational School Gumushane University Gumushane Turkey

**Keywords:** estimated daily intake, hazard index, health risk, heavy metal, traditional cheese

## Abstract

Traditional cheeses from Turkey's Black Sea region, such as Aho, Golot, and Telli, are widely known for their unique flavors and artisanal production methods. However, their microbiological safety, mineral content, and heavy metal contamination remain understudied. In this study, we evaluated 30 cheese samples for essential minerals and toxic heavy metals, alongside conducting a corresponding health risk assessment. The results of our mineral analysis revealed sodium (Na) levels ranging from 9787.79 to 37,902.22 mg/kg, while calcium (Ca) and magnesium (Mg) were present at nutritionally relevant concentrations. Heavy metals such as lead (Pb) and mercury (Hg) were detected, with Golot and Telli cheeses showing elevated levels (Pb: up to 1788.75 μg/kg; Hg: up to 468.71 μg/kg). Furthermore, health risk assessments, including estimated daily intake (EDI), target hazard quotient (THQ), and hazard index (HI), indicated that 93.3% of samples posed negligible risks (HI < 1), though two outliers (6.7%) exceeded safety thresholds. Taken together, these findings underscore the need for improved production processes in traditional cheese production while affirming their nutritional value and overall safety for consumers.

## Graphical abstract

Elemental composition and health risk assessment of traditional Trabzon cheeses.

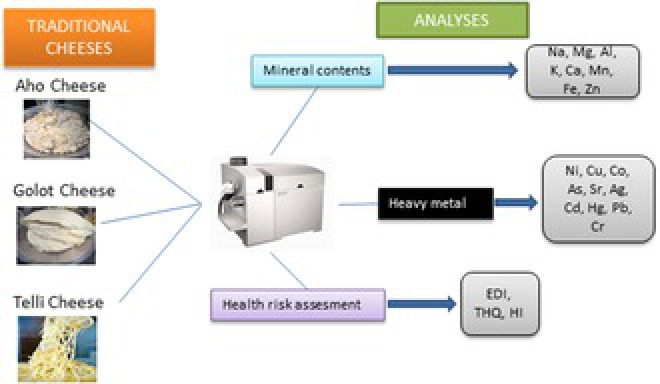

## 1 Introduction

Turkey offers a rich variety of dairy products, including many industrial and traditional cheeses. The most well‐known cheeses nationwide include White, Kashar, Tulum, Çökelek, and Lor (Temiz and Kiliç 2016). Overall, there are different cheeses, about 150–200 in Turkey (Hacıoglu and Kunduhoglu 2021). The production process and components of cheeses could indicate a difference based on the geographic region. These discrepancies directly affect the nutrient value, mineral content, and microbiological quality of cheeses (Kamber 2015; Kırdar 2024; Ulusoy et al. 2024).

The Black Sea Region is known for a variety of traditional dairy products. In the region, Aho, Golot, and Telli cheeses have a conventional production process and a specific aroma (Dinkci et al. 2012; Kamber and Terzi 2008; Sekban and Tarakci 2020). However, available research on these cheeses' microbiological quality, mineral content, heavy metals, and health risk assessment is limited. The mineral content is essential for human health, while heavy metals could be harmful (Kalaycı et al. 2023). Similar concerns about microbial diversity and biogenic amine formation were documented in other fermented foods, such as sufu, where post‐fermentation conditions significantly impact safety (Guo et al. 2025). This underscores the need to evaluate microbiological risks in traditional cheeses.

Minerals are essential for enzymatic functions, hormonal balance, and regulatory functions (Beikzadeh et al. 2019; Ergin et al. 2021). However, milk and dairy products could accumulate heavy metals such as lead (Pb), mercury (Hg), arsenic (As), and cadmium (Cd) in the human body. This could lead to serious health problems in humans, such as kidney failure, genetic mutations, nervous system disorders, cardiovascular disorders, and several cancer types (Beikzadeh et al. 2019; Al Sidawi et al. 2021). Similarly to heavy metals, other contaminants like mycotoxins (e.g., aflatoxin M1) in dairy products were reported to pose significant health risks to consumers (Xiong et al. 2022). This highlights the need for comprehensive safety evaluations of traditional dairy products.

Furthermore, in terms of traditional cheese varieties, Turkey's top three regions are the Black Sea (100 varieties), the Mediterranean (72 varieties), and the Aegean Region. Among these, Trabzon Province has the highest number of distinct cheeses—21 varieties (Kırdar 2024). However, despite the nutritional importance of these cheeses, there remain challenges in cheese production associated with the lack of legal rules, a standard production process, and suitable production equipment (Kırdar et al. 2013; Özlü et al. 2012). In this context, the present study aims to investigate microbiological quality, mineral content, and heavy metal contamination in Aho, Golot, and Telli cheeses collected from Trabzon, Turkey, as well as to evaluate health risks associated with these cheese consumption.

## 2 Materials and Methods

### 2.1 Material

Samples of Aho, Golot, and Telli cheeses were collected from local markets in April and May in Trabzon, Turkey. The local markets were randomly selected from among those selling traditional products. Of each of the three cheeses, 10 samples weighing about 250 g were collected. The samples were placed into sterile jars and transferred by cold chain to the laboratory, where they were stored in the refrigerator and freezer until subsequent analysis.

### 2.2 Mineral and Heavy Metal Analysis

Mineral and heavy metal analyses were determined using the wet incineration method (Güler 2007). The 2 g cheese sample was placed into the vessels of microwave (Start D, Milestone Inc., Sorisole, Italy). Next, HNO_3_ (8 mL; 65%) and H_2_O_2_ (2 mL; 30%) were added to the samples for digestion. For the microwave process, the samples were heated up to 200°C for 15 min, kept at 45 min and 500 bar, and chilled afterward. The mineral and heavy metal content of the samples was diluted to 100 mL with ultrapure water. The contents were determined by ICP‐MS (Agilent 7700, Japan). Validation data of mineral and heavy metal analysis are summarized in Table 1.

**TABLE 1:** Validation values of mineral and heavy metals.

Parameters	Calibration curve	*R* ^2^	LOD (μg/L)	LOQ (μg/L)
Na	*y* = 999681.0189*x* + 33034.7533	0.9993	0.095	0.316
Ca	*y* = 8634.7144*x* + 80.0033	0.9999	0.121	0.404
Mg	*y* = 413488.5920*x* + 22229.68	0.9999	0.043	0.143
K	*y* = 336926.5765*x* + 52111.3667	0.9999	0.062	0.206
Al	*y* = 120.1132*x* + 100.0033	0.9997	0.119	0.396
Mn	*y* = 3554.9345*x* + 436.6933	1.0000	0.214	0.713
Fe	*y* = 6334.0076*x* + 5921.5233	0.9999	0.478	1.593
Zn	*y* = 1448.2591*x* + 3537.2467	0.9998	0.152	0.506
Ni	*y* = 4059.3941*x* + 11302.5167	1.0000	0.058	0.193
Cu	*y* = 10928.1928*x* + 1636.8100	0.9996	0.045	0.151
Co	*y* = 15629.2719*x* + 80.0033	0.9994	0.166	0.554
As	*y* = 1188.6002*x* + 7.667	0.9995	0.4887	1.629
Sr	*y* = 3769.1537*x* + 293.3500	1.0000	0.094	0.314
Ag	*y* = 37278.0143*x* + 330.0200	0.9999	0.045	0.151
Cd	*y* = 7454.6653*x* + 23.3333	0.9999	0.037	0.125
Hg	*y* = 22.1890*x* + 28.3333	0.9998	0.033	0.173
Pb	*y* = 38732.1674*x* + 496.6967	1.0000	0.063	0.210
Cr	*y* = 8057.88714*x* + 241.3333	0.9998	0.074	0.246

**TABLE 2:** Mineral content of cheese samples (mg/kg).

Parameters		Aho cheese (*n* = 10)	Golot cheese (*n* = 10)	Telli cheese (*n* = 10)	Sig.
Na	Min	30728.49	555.28	4717.64	**
	Max	50620.09	38375.68	12252.36	
	Mean	37902.66 ± 5997.87a	20826.18 ± 12603.13b	9787.79 ± 2125.38c	
	95% CI	33612.04–42193.28	11810.44–29841.91	8267.38–11308.19	
Ca	Min	311.68	166.63	789.99	NS
	Max	1260.27	1407.97	1257.21	
	Mean	771.42 ± 287.47ab	644.83 ± 395.32b	963.70 ± 144.53a	
	95% CI	565.78–977.07	362.04–927.62	860.31–1067.09	
Mg	Min	234.24	174.92	242.89	NS
	Max	446.24	349.51	449.98	
	Mean	333.09 ± 64.80ab	274.90 ± 58.79b	340.75 ± 69.11a	
	95% CI	286.73–379.45	232.84–316.96	291.31–390.18	
K	Min	1012.89	750.84	564.53	*
	Max	1398.32	1113.68	1488.69	
	Mean	1142.85 ± 116.43a	922.83 ± 121.67b	973.67 ± 253.06b	
	95% CI	1059.06–1226.14	835.80–1009.87	792.63–1154.70	
Al	Min	2.31	0.85	0.93	*
	Max	34.63	34.55	0.70	
	Mean	16.57 ± 11.82a	10.01 ± 12.20ab	2.53 ± 1.95b	
	95% CI	8.11–25.03	1.28–18.73	1.13–3.92	
Mn	Min	0.10	0.10	0.18	**
	Max	0.49	0.37	0.89	
	Mean	0.30 ± 0.12ab	0.22 ± 0.10b	0.43 ± 0.27a	
	95% CI	0.21–0.38	0.15–0.29	0.24–0.62	
Fe	Min	2.39	0.93	1.20	NS
	Max	11.79	15.63	6.44	
	Mean	6.00 ± 3.06a	5.66 ± 5.16a	3.21 ± 1.93a	
	95% CI	3.81–8.19	1.97–9.35	1.83–4.59	
Zn	Min	6.01	24.67	32.58	**
	Max	29.08	76.57	53.51	
	Mean	18.24 ± 9.19b	45.86 ± 18.20a	43.04 ± 7.00a	
	95% CI	11.67–24.81	32.85–58.88	38.04–48.05	

*Note:* Different small letters (a, b, c) indicate statistical significance for mean values between the samples (*p* < 0.05).

Abbreviations: 95% CI: 95% confidence interval; NS: nonsignificant.

* *p* < 0.05.

** *p* < 0.01.

**TABLE 3:** EDI values of mineral content of cheese samples (mg/kg BW per day).

Parameters		Aho cheese (*n* = 10)	Golot cheese (*n* = 10)	Telli cheese (*n* = 10)
Na	Min	23.57	0.43	3.62
	Max	38.83	29.44	9.40
	Mean	29.08 ± 4.60	15.98 ± 9.67	7.51 ± 1.63
	95% CI	28.78–32.37	9.06–22.89	6.34–8.67
Ca	Min	0.24	0.13	0.61
	Max	0.97	1.08	0.96
	Mean	0.59 ± 0.22	0.49 ± 0.30	0.74 ± 0.11
	95% CI	0.43–0.75	0.28–0.71	0.66–0.82
Mg	Min	0.18	0.13	0.19
	Max	0.34	0.27	0.35
	Mean	0.26 ± 0.05	0.21 ± 0.05	0.26 ± 0.05
	95% CI	0.22–0.29	0.18–0.24	0.22–0.30
K	Min	0.78	0.58	0.43
	Max	1.07	0.85	1.14
	Mean	0.88 ± 0.09	0.71 ± 0.09	0.75 ± 0.19
	95% CI	0.81–0.094	0.64–0.77	0.61–0.89
Al	Min	0.002	0.001	0.001
	Max	0.027	0.027	0.005
	Mean	0.013 ± 0.009	0.008 ± 0.009	0.002 ± 0.001
	95% CI	0.006–0.019	0.001–0.014	0.001–0.003
Mn	Min	0.0001	0.0001	0.0001
	Max	0.0004	0.0003	0.0007
	Mean	0.0002 ± 0.0001	0.0002 ± 0.0001	0.0003 ± 0.0002
	95% CI	0.0002–0.0003	0.0001–0.0002	0.0002–0.0005
Fe	Min	0.002	0.001	0.001
	Max	0.009	0.012	0.005
	Mean	0.005 ± 0.002	0.004 ± 0.004	0.002 ± 0.001
	95% CI	0.003–0.006	0.002–0.007	0.001–0.004
Zn	Min	0.005	0.019	0.025
	Max	0.022	0.059	0.041
	Mean	0.014 ± 0.007	0.035 ± 0.014	0.033 ± 0.005
	95% CI	0.009–0.019	0.002–0.045	0.029–0.037

Abbreviations: 95% CI: 95% confidence interval; EDI: estimated daily intake of metals.

### 2.3 Health Risk Assessment

#### 2.3.1 Estimated Daily Intake (EDI)

For each of the samples, EDI values of the heavy metals and minerals were determined using Equation (1) (Rocha et al. 2023):

(1) $$\mathrm{EDI}=\frac{C_{\text{metal}\times W_{\text{cheese}}}}{\mathrm{BW}}$$

where *C*_*metal*_ is the concentration of heavy metals and minerals in the cheese samples, *W*_*cheese*_ is the daily average consumption of cheese (kg; assumed to be 19.6 kg a year according to the Ministry of Agriculture and Forestry of the Turkish Republic; Anonim 2023). Finally, BW refers to body weight (assumed to be 70 kg), while RDA is the recommended dietary allowance for minerals, and is given in Table 4.

**TABLE 4:** Contribution to daily intake of mineral content of cheese samples (%).

Parameters		Aho cheese (*n* = 10)		Golot cheese (*n* = 10)		Telli cheese (*n* = 10)		RDA (mg/day)
		Man	Woman	Man	Woman	Man	Woman	
Na	Min	1.57		0.03		0.24		1500 (National Institutes of Health, 2024)
	Max	2.59		1.96		0.63		
	Mean	1.94		1.07		0.50		
Ca	Min	0.02		0.01		0.06		1000–1200 (National Institutes of Health 2024)
	Max	0.10		0.11		0.10		
	Mean	0.06		0.05		0.07		
Mg	Min	0.05	0.06	0.03	0.04	0.05	0.06	400–420 for men and 310–320 for women (National Institutes of Health 2024)
	Max	0.09	0.11	0.07	0.09	0.09	0.11	
	Mean	0.07	0.08	0.05	0.07	0.07	0.08	
K	Min	0.02	0.03	0.02	0.02	0.01	0.02	3400 for men and 2600 for women (National Institutes of Health 2024)
	Max	0.03	0.04	0.03	0.03	0.03	0.04	
	Mean	0.03	0.03	0.02	0.03	0.02	0.03	
Al	Min							No information
	Max							
	Mean							
Mn	Min	0.00	0.01	0.00	0.01	0.00	0.01	2.3 for men and 1.8 for women (National Institutes of Health 2024)
	Max	0.02	0.02	0.01	0.02	0.03	0.04	
	Mean	0.01	0.01	0.01	0.01	0.01	0.02	
Fe	Min	0.11	0.03	0.06	0.01	0.06	0.01	8 for men and 18 for women (National Institutes of Health 2024)
	Max	0.50	0.11	0.67	0.15	0.28	0.06	
	Mean	0.28	0.06	0.22	0.05	0.11	0.03	
Zn	Min	0.05	0.06	0.17	0.24	0.23	0.31	11 for men and 8 for women (National Institutes of Health 2024)
	Max	0.20	0.28	0.54	0.74	0.37	0.51	
	Mean	0.13	0.18	0.32	0.44	0.30	0.41	

Abbreviation: RDA: recommended dietary allowance.

**TABLE 5:** Heavy metal content of cheese samples (μg/kg).

Parameters		Aho cheese (*n* = 10)	Golot cheese (*n* = 10)	Telli cheese (*n* = 10)	Significance
Ni	Min	ND	ND	ND	NS
	Max	276.40	3681.67	664.88	
	Mean	154.13 ± 172.91a	1502.47 ± 1887.35a	416.06 ± 352.63a	
	95% CI	0.00–1707.65	0.00–6190.91	0.46–1292.04	
Cu	Min	318.44	221.36	280.28	NS
	Max	698.55	8374.00	773.94	
	Mean	521.09 ± 94.97a	1300.73 ± 2489.81a	456.26 ± 164.30a	
	95% CI	453.16–589.03	0.00–3081.84	338.72–573.79	
Co	Min	2.53	1.34	2.39	NS
	Max	5.29	7.81	13.77	
	Mean	3.78 ± 1.04a	3.46 ± 1.98a	6.16 ± 4.94a	
	95% CI	3.04–4.53	2.04–4.88	2.62–9.69	
As	Min	ND	ND	ND	NS
	Max	8.90	3.53	6.17	
	Mean	3.54 ± 2.65a	2.51 ± 1.00a	4.71 ± 0.91a	
	95% CI	1.50–5.57	1.46–3.57	3.58–5.84	
Sr	Min	5264.27	2085.09	4254.16	NS
	Max	9428.36	11340.26	8311.94	
	Mean	7377.10 ± 1383.80a	5828.87 ± 3055.11a	5754.44 ± 1330.18a	
	95% CI	6387.19–8367.01	3643.38–8014.36	4802.88–6705.99	
Ag	Min	1.27	0.38	6.74	**
	Max	24.12	8.43	18.96	
	Mean	6.78 ± 6.62b	4.01 ± 2.29b	12.70 ± 4.08a	
	95% CI	2.05–11.52	2.37–5.65	9.79–15.62	
Cd	Min	0.57	ND	ND	NS
	Max	7.07	10.66	9.82	
	Mean	2.08 ± 2.16a	3.58 ± 3.38a	3.28 ± 3.42a	
	95% CI	0.53–3.63	0.98–6.17	0.65–5.91	
Hg	Min	ND	ND	ND	**
	Max	ND	ND	468.71	
	Mean	ND	ND	154.55 ± 210.23a	
	95% CI	—	—	0.00–165.74	
Pb	Min	29.17	10.58	16.17	NS
	Max	108.25	1788.75	108.00	
	Mean	56.54 ± 23.68a	241.87 ± 546.69a	48.08 ± 25.75a	
	95% CI	39.60–73.49	0.00–632.95	29.65–66.50	
Cr	Min	23.24	17.36	29.97	NS
	Max	300.76	341.95	274.79	
	Mean	67.90 ± 83.41a	78.03 ± 95.74a	85.43 ± 72.47a	
	95% CI	8.23–127.57	9.54–146.51	33.59–137.27	

*Note:* Different small letters (a, b, c) indicate statistical significance for mean values between the samples (*p* < 0.05).

Abbreviations: 95% CI: 95% confidence interval; ND: not detected; NS: nonsignificant.

** *p* < 0.01.

#### 2.3.2 Target Hazard Quotient (THQ)

THQ quantifies the noncarcinogenic health risk associated with exposure to a toxic compound. A THQ value of 1 or greater indicates a potential health concern, while a value below 1 suggests that the health risk is negligible (Basaran and Turk 2024). THQ was calculated using Equation (2).

(2) $$\mathrm{THQ}=\frac{\mathrm{EDI}}{\mathrm{RfD}}$$

where *EDI* represents the estimated daily metal exposure (μg/kg BW/day), while *RfD* indicates oral reference dose and is provided in Table 6.

**TABLE 6:** EDI values of heavy metal content of cheese samples (μg/kg BW per day).

Parameters		Aho cheese (*n* = 10)	Golot cheese (*n* = 10)	Telli cheese (*n* = 10)	RfD (mg/day)
Ni	Min	—	—	—	0.02 (EPA 2024)
	Max	0.21	2.82	0.51	
	Mean	0.12 ± 0.07	1.15 ± 0.88	0.32 ± 0.20	
	95% CI	0.00–0.07	0.00–0.98	0.00–0.24	
Cu	Min	0.24	0.17	0.22	0.04 (EPA 2024)
	Max	0.54	6.42	0.59	
	Mean	0.40 ± 0.07	1.00 ± 1.91	0.35 ± 0.13	
	95% CI	0.035–0.045	0.00–2.36	0.26–0.44	
Co	Min	0.002	0.001	0.002	0.0003 (EPA 2024)
	Max	0.004	0.006	0.011	
	Mean	0.003 ± 0.001	0.003 ± 0.002	0.005 ± 0.004	
	95% CI	0.002–0.003	0.002–0.004	0.002–0.007	
As	Min	—	—	—	0.0003 (EPA 2024)
	Max	0.007	0.003	0.005	
	Mean	0.003 ± 0.002	0.002 ± 0.001	0.004 ± 0.002	
	95% CI	0.001–0.004	0.000–0.0002	0.000–0.0003	
Sr	Min	4.04	1.60	3.26	0.06 (EPA 2024)
	Max	7.23	8.70	6.38	
	Mean	5.66 ± 1.06	4.47 ± 2.34	4.41 ± 1.02	
	95% CI	4.90–6.42	2.79–6.15	3.68–5.14	
Ag	Min	0.001	0.000	0.005	0.005 (EPA 2024)
	Max	0.019	0.006	0.015	
	Mean	0.005 ± 0.005	0.003 ± 0.002	0.010 ± 0.003	
	95% CI	0.002–0.009	0.002–0.004	0.008–0.012	
Cd	Min	0.000	—	—	0.0001 (EPA 2024)
	Max	0.005	0.008	0.008	
	Mean	0.002 ± 0.002	0.003 ± 0.003	0.003 ± 0.003	
	95% CI	0.000–003	0.001–0.004	0.000–0.004	
Hg	Min	—	—	—	0.0003 (EPA 2024)
	Max	—	—	0.360	
	Mean	—	—	0.047 ± 0.111	
	95% CI	—	—	0.00–0.13	
Pb	Min	0.02	0.01	0.01	0.0005 (EPA 2024)
	Max	0.08	1.37	0.08	
	Mean	0.04 ± 0.02	0.19 ± 0.42	0.04 ± 0.02	
	95% CI	0.03–0.06	0.00–0.49	0.02–0.05	
Cr	Min	0.02	0.01	0.02	0.003 (EPA 2024)
	Max	0.23	0.26	0.21	
	Mean	0.05 ± 0.06	0.06 ± 0.07	0.07 ± 0.06	
	95% CI	0.01–0.10	0.01–0.11	0.03–0.11	

Abbreviations: 95% CI: 95% confidence interval; RfD: oral reference dose.

#### 2.3.3 Hazard Index

The target hazard quotient (THQ) represents an important risk assessment tool to evaluate potential health effects from dietary exposure to chemically contaminated foods (Rocha et al. 2023). The hazard index (HI) represents the cumulative sum of the individual THQs for various elements evaluated across different food types. This index assumes that consuming a specific food type may lead to simultaneous exposure to multiple potentially harmful compounds. Consequently, the combined effects of these toxic substances could result in negative health outcomes. An HI below 1 indicates no health concern; an HI value between 1 and 5 reflects a low level of health concern, while an HI ranging from 10 to 100 signifies a significant health concern, warranting systematic monitoring (Basaran 2022; Basaran and Turk 2024). The HI is calculated using Equation (3).

(3) HI=∑THQ

### 2.4 Statistical Analyses

The SPSS 21 software package was used for the statistical evaluation of the data. One‐way ANOVA and Duncan's multiple‐comparison test were employed to identify significant differences, except for EDI, THQ, and HI values. The significance level was set at *p* < 0.05. The homogenized test was performed on these data. The maximum, minimum, mean, and standard deviation were determined for EDI, THQ, and HI values.

## 3 Results and Discussion

### 3.1 Mineral Content

#### 3.1.1 Sodium (Na)

**FIGURE 1:**
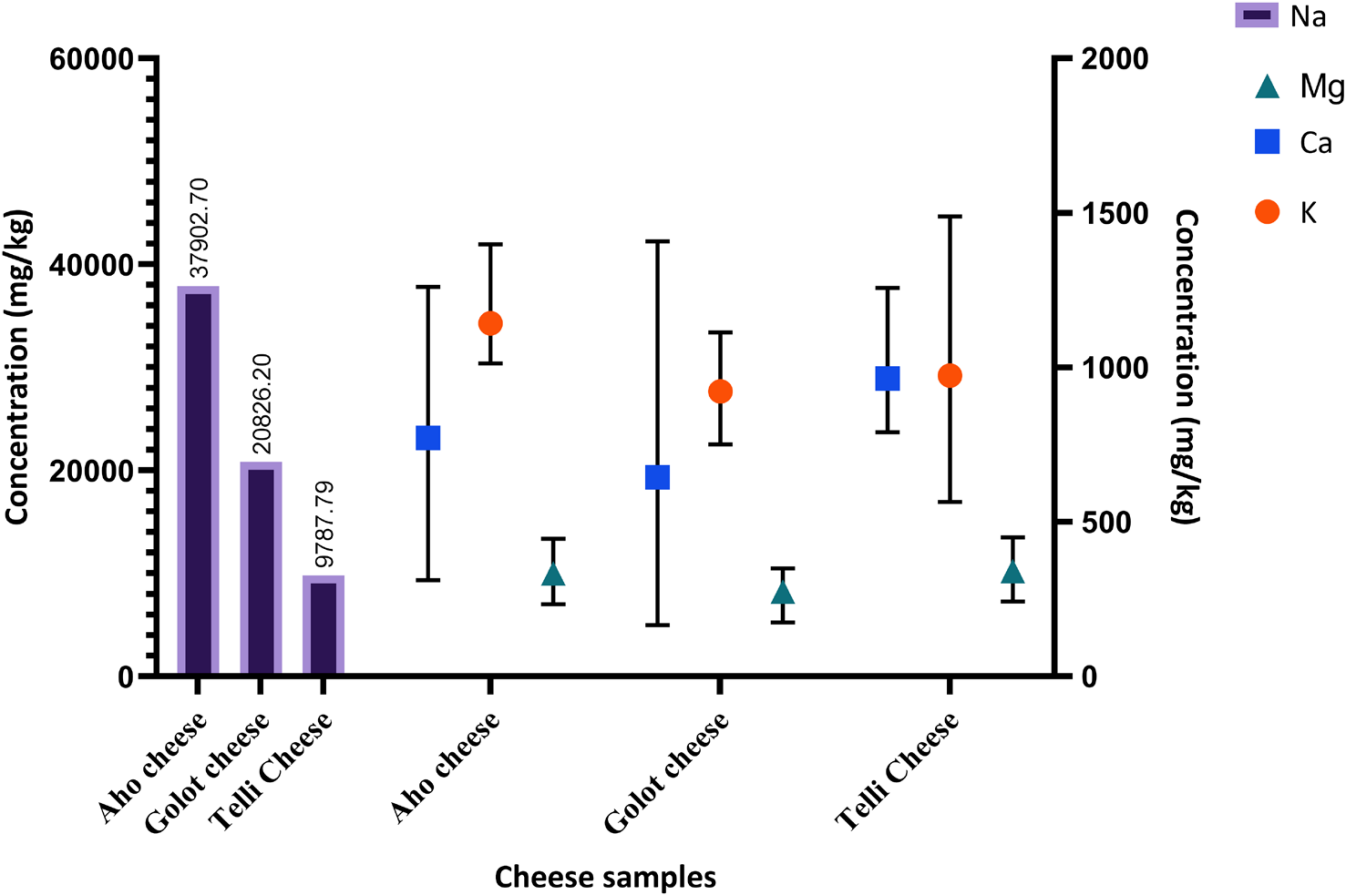
Na, Mg, Ca, and K contents of the cheese samples.

The mineral amounts of cheese samples are reported in Table 2. The Na content was not affected by the type of cheese (*p* < 0.01). The Na mean amount was between 9787.79 and 37902.22 mg/kg. The lowest mean of Na concentration was in the Telli cheese, while the highest value was in the Aho cheese (see Figure 1). The lowest, highest, and Na mean contents of traditional Çökelek cheese were found to be 22.8, 9035, and 941 mg/kg, respectively (Kalaycı et al. 2023). However, previous studies reported lower values. For instance, Kirdar et al. (2015) determined that the Na values of Kargı Tulum cheese changed between 1153.0 and 1850.3 mg/kg during storage. Mendil (2006) found the Na amounts of different traditional cheese samples collected in Turkey between 3957 and 6558 μg/g.

The dietary intake of metals is determined by two key factors: the concentration of metals in food products and the quantity of those foods consumed daily. Furthermore, an individual's body weight plays a critical role in their physiological tolerance to contaminants (Meshref et al. 2014). The EDI values of Na were 0.43 and 38.83 mg/kg BW per day (Table 3), and the mean contribution to daily intake ranged between 0.50% and 2.59% (Table 5). Aho cheese had the highest contribution ratio for Na.

#### 3.1.2 Calcium (Ca)

The Ca contents were not significantly affected by the variety of cheese (*p* > 0.05). The Ca amounts of the Aho, Golot, and Telli cheeses ranged 311.68–1260.27 mg/kg, 166.63–1407.97 mg/kg, and 789.99–1257.21 mg/kg, respectively (see Figure 1). The Golot cheese had a lower mean of Ca content compared to other samples (Table 2). The calcium content of mozzarella cheeses was determined to be between 7316.72 and 9421.10 mg/kg (Capcarova et al. 2024). Kalaycı et al. (2023) found that the Ca quantity of Çökelek cheese sold in Trabzon changed between 19.3 and 714 mg/kg. Another relevant study found that the Ca amount of traditional Malatya cheese ranged from 3237.89 to 7081.07 mg/kg (Kose et al. 2022). The obtained results were lower than those reported by Capcarova et al. (2024) and Kose et al. (2022) and higher than those reported by Kalaycı et al. (2023).

The average EDI values of Ca changed from 0.96 to 0.74 mg/kg BW per day (Table 3). The RDA of Ca is between 1000 and 1200 mg/day (Table 4). The contribution to EDI for Ca was the lowest (0.01%) and the highest (0.11%) in the Golot cheese samples.

#### 3.1.3 Magnesium (Mg)

The various types of cheese did not have statistically significant (*p* > 0.05) effect on the Mg rate. The highest Mg mean ratio was in the Telli cheese samples (Table 2). The Mg amount of all cheeses was between 174.92 mg/kg (Golot) and 449.98 mg/kg (Telli) (Figure 1). The Mg contents of different studies were found between 282.42 and 378.01 mg/kg in mozzarella (Capcarova et al. 2024), 0.16 and 0.24 mg/g in Örgü cheese (Çelebi and Şimşek 2015), and 92.12 and 161.73 mg/kg in Çökelek samples (Öksüztepe et al. 2013). The results observed herein were higher than those determined by Capcarova et al. (2024), Çelebi and Şimşek (2015), and Öksüztepe et al. (2013).

The EDI and contribution ratio are provided in Tables 2 and 3. The Telli and Aho cheeses have exhibited the same average EDI values for Mg (0.26 mg/kg BW per day), while Golot cheese had a lower EDI value (0.21 mg/kg BW per day). The RDA of Ca is 400–420 mg/day for men and 310–320 mg/day for women (Table 4). The average contribution values of Mg for men and women were 0.05%–0.07% and 0.07%–0.08%, respectively.

#### 3.1.4 Potassium (K)

The results concerning K amounts are reported in Table 2. The K content significantly varied among the various types of cheeses (*p* < 0.05). The Telli cheese samples had the lowest (564.53 mg/kg) and highest (1488.69 mg/kg) K amounts (see Figure 1). The K mean content of Aho cheese was higher than other samples. Kalaycı et al. (2023) determined that the mean Mg content of the traditional Çökelek cheeses was 72.6 mg/kg, while Kose et al. (2022) found the K content of 644.29 mg/kg in the traditional Malatya cheeses. In another relevant study, the lowest, highest, and K mean values of Çökelek samples were 452.14, 2462.40, and 1142.24 mg/kg, respectively (Öksüztepe et al. 2013). The findings presented by Kalaycı et al. (2023) exhibited lower values compared to the results obtained in this study. Conversely, the results reported by Kose et al. (2022) demonstrated higher values than those obtained in this research.

The Telli cheese had the lowest and highest EDI values for K (see Table 3). The mean contribution to daily intake for both men and women ranged between 0.02% and 0.03% (Table 4).

#### 3.1.5 Aluminum (Al)

The Al content significantly varied across the three tested cheese types (*p* < 0.05). The lowest and highest Al amounts were 0.85 mg/kg (Golot) and 34.63 mg/kg (Aho), respectively (Table 2). The differences in the mean Al rates of samples were statistically significant (*p* < 0.05). The Al quantities in previous studies were detected as 0.10–0.15 mg/kg in mozzarella cheese (Arella et al. 2024) and 0.000–134 mg/kg in Çökelek cheese (Kalaycı et al. 2023). Therefore, results determined in the present study were higher than those reported by Capcarova et al. (2024) and Kalaycı et al. (2023).

The EDI mean values of Al changed between 0.002 and 0.013 mg/kg BW per day (see Table 3). No information on the recommended dietary allowance of Al is available.

#### 3.1.6 Manganese (Mn)

The type of cheese had a significant effect (*p* < 0.01) on the Mn amounts in the samples (Table 2). The minimum and maximum Mn values were 0.10 and 0.89 mg/kg, respectively. The mean Mn value of the Golot cheese samples was lower than compared of other samples. Kirdar et al. (2015) found that Mn quantities of Kargi Tulum cheese samples were between 0.11 and 0.19 mg/kg during storage. In another relevant study, Çelebi and Şimşek (2015) reported that Mn was not detected in the traditional Örgü cheeses. Mendil (2006) determined the Mn contents of different types of traditional cheese to be between 0.28 and 1.1 mg/kg. The results determined by Mendil (2006) were higher as compared with the results of the present study, while those reported by Kirdar et al. (2015) and Çelebi and Şimşek (2015) were lower.

The mean EDI values of Mn were identical for Aho and Golot cheeses, whereas Telli cheese differed (see Table 3). For men, the mean contribution to daily intake was 0.01%, while the ratios for women ranged from 0.01% to 0.02% for Mn (Table 4).

#### 3.1.7 Iron (Fe)

The Fe amounts significantly differed among the three tested cheese varieties (*p* > 0.05). The Golot cheeses had the minimum (0.93 mg/kg) and maximum (15.63 mg/kg) Fe content (see Table 2). The mean of Fe values was between 3.21 and 6.00 mg/kg. However, there were no statistically significant changes in the Fe amounts of samples (*p* > 0.05). Similarly, in previous research, the Fe amounts were detected to amount to 3.81–11.68 mg/kg in traditional Malatya cheeses (Kose et al. 2022), 1.7633–14.7388 ppm in Kareish cheese (Meshref et al. 2014), and 7.49–29.05 μg/g in Akçakatık cheese (Kırdar et al. 2013).

The EDI values for Fe ranged from 0.001 to 0.012 mg/kg BW per day (see Table 3). The RDA values were 8 and 18 mg/day for women (see Table 4). The mean contribution to daily intake for Fe for men and women ranged between 0.11%–0.28% and 0.03%–0.06%, respectively (Table 4).

#### 3.1.8 Zinc (Zn)

The Zn content significantly differed across the three studied cheese varieties (*p* < 0.01) (see Table 2). The Zn values changed from 6.01 to 32.58 mg/kg. The Zn mean value of the Aho cheese sample was lower than that of the other cheese samples. These results were similar to those determined by Kırdar et al. (2013), who studied traditional Akçakatık cheese, and by Meshref et al. (2014), who studied traditional Kareish cheese (Egypt). However, the results obtained in this study were higher than the determined result (0.03 mg/kg) by Çelebi and Şimşek (2015).

The Aho cheese had the highest average EDI amount for Zn, whereas the Golot cheese was the lowest (see Table 3). The contribution to EDI amounts for Zn ranged between 0.13% and 0.30% for men and 0.18% and 0.44% for women (Table 4).

### 3.2 Heavy Metal Content

#### 3.2.1 Nickel (Ni)

The Ni contents in the samples were not significantly different depending on the type of cheese (Table 5). The Ni amount was determined in only 2 out of 10 Aho cheeses, 3 out of 10 Golot cheeses, and 3 out of 10 Telli cheeses. There were no significant changes between Ni mean amounts (see Table 5). Ni amounts of mozzarella cheese were determined to range between 0.72 and 1.61 mg/kg by Capcarova et al. (2024). Al Sidawi et al. (2021) reported that Ni values of Imeruli and Sulguni samples (Georgian traditional cheese) were 0.011 and 0.026 mg/kg, respectively. In another study, Ni contents in different traditional types of cheese collected changed between 0.18 and 0.34 μg/g, as reported by Mendil (2006). The mean values observed in the present study were significantly higher than those reported by Al Sidawi et al. (2021) and Mendil (2006) and lower than those by Capcarova et al. (2024). However, Öksüztepe et al. (2013) reported that the Ni contents were not detected in Çökelek samples.

Possible reasons for the elevated concentrations may include the use of temporary storage tanks and contaminated milking equipment during initial milk collection and processing. In addition, contamination could arise from certain raw materials in animal feed, as nickel (Ni) is sometimes used as a catalyst in feed production. However, animals absorb only a small portion of ingested Ni, with most of it being excreted—primarily through urine and, to a lesser extent, through milk (Christophoridis et al. 2019). Overall, Ni exposure adversely affects the reproductive and immune systems and may induce oxidative stress and genotoxicity. However, no conclusive evidence currently links oral nickel exposure to cancer in humans (Schrenk et al. 2020).

#### 3.2.2 Copper (Cu)

The Cu quantities did not significantly differ across the three cheese varieties. The Golot cheese exhibited a minimum (221.36 μg/kg) and a maximum (8374.73 μg/kg) Cu values (see Table 5). The mean Cu contents ranged from 521.09 to 1300.73 μg/kg. Meshref et al. (2014) determined Cu contents in traditional Egyptian cheese (Kareish) to range between 0.0036 and 0.9312 ppm, with a mean of 0.953 ppm. Furthermore, Çelebi and Şimşek (2015) found that Cu amounts of Örgü cheeses changed between 0.001 mg/kg and 0.006 at the first day of storage, while Cu quantities were not detected at the end of storage. In another relevant study, Kırdar et al. (2013) reported that Cu values of Akçakatık cheese ranged from 1.44 to 8.69 μg/g. The Cu results determined in the previous studies were lower than those observed in the present study.

The elevated copper content could be attributed to contamination from containers during the transportation and processing of cheese milk (Dağcilar and Gezer 2021; Kirdar et al. 2015). Copper (Cu) is an essential micronutrient that plays a vital role in numerous physiological processes such as growth, cardiovascular function, lung elasticity, neuroendocrine regulation, and iron metabolism. However, excessive copper intake can induce adverse health effects, such as gastrointestinal disturbances, immune dysfunction, liver cirrhosis, neurological disorders, and dermatitis (Kalaycı et al. 2023; Meshref et al. 2014; Al Sidawi et al. 2021).

#### 3.2.3 Cobalt (Co)

The amount of Co did not significantly differ across the three tested cheese varieties (see Table 5). The Co amounts of samples ranged between 1.34 μg/kg (Golot cheese) and 13.77 μg/kg (Telli cheese). In previous research, Co contents were found to amount to 0.013 mg/kg (Imeruli cheese) and 0.03 mg/kg (Sulguni cheese) in Georgian traditional cheeses (Al Sidawi et al. 2021) and 20–154 μg/100 g in white cheeses produced in Diyarbakır (Türkiye) (Merdivan et al. 2004). These results were lower than those reported by Al Sidawi et al. (2021) and Merdivan et al. (2004).

The elevated cobalt content in milk and dairy products primarily results from bovine metabolic processes, as certain metals (including cobalt) can undergo direct transfer from blood to milk (Al Sidawi et al. 2021). The primary biological role of Co lies in its essential function as the central atom in the vitamin B12 (cobalamin) coenzyme complex (Basaran and Turk 2024). While cobalt is an essential trace element, its excessive consumption can lead to toxicological effects that can induce neurological impairments, thyroid dysfunction, and polycythemia (Al Sidawi et al. 2021).

#### 3.2.4 Arsenic (As)

The As contents were not detected in 1 of 10 Aho cheese, 4 of 10 Golot cheese, and 5 of 10 Telli cheese. The mean As values in Aho, Golot, and Telli cheeses were 3.54, 2.51, and 4.71 μg/kg, respectively. However, these differences were not statistically significant (*p* > 0.05) (Table 5). Similarly, Capcarova et al. (2024) did not detect arsenic in mozzarella cheeses. Kalaycı et al. (2023) found that the mean As content was 0.038 mg/kg in Çökelek cheeses. Dağcilar and Gezer (2021) determined As content to be 0.66 μg/kg in Lor cheeses. The results of the present study are partly consistent with those reported by Capcarova et al. (2024) and were lower than those reported by Kalaycı et al. (2023) and Dağcilar and Gezer (2021). Conversely, Öksüztepe et al. (2013) found that the As contents in Çökelek samples were not detected. Similarly, Kalaycı et al. (2023) reported that some Çökelek (26 of 30 samples) cheeses did not have the As amount.

Elevated arsenic (As) levels may originate from intensive agricultural practices involving pesticides, herbicides, and insecticides (Christophoridis et al. 2019; Özlü et al. 2012). Furthermore, groundwater and drinking water contaminated with high As concentrations can contribute to elevated As levels in dairy products (Dağcilar and Gezer 2021). Other possible factors include milk production conditions and processing equipment (Kalaycı et al. 2023). The high As contamination was previously reported to exhibit carcinogenic properties while compromising immune function, thereby increasing disease vulnerability and demonstrating significant cardiotoxicity and neurotoxicity at elevated exposure levels (Basaran and Turk 2024).

#### 3.2.5 Strontium (Sr)

The Sr amounts were not affected by the type of cheese (*p* > 0.05). The lowest and highest Sr contents were identified in the Golot cheese samples (Table 5). The mean Sr amounts of samples ranged from 5754.44 to 7377.10 μg/kg. Capcarova et al. (2024) found that the Sr values of mozzarella cheeses sold in the Slovak market ranged from 3.37 to 3.69 mg/kg. The results observed in the present study were higher than those reported by Capcarova et al. (2024).

Road dust from heavily trafficked areas and intensive agricultural fertilizer use serve as significant sources of strontium (Sr) pollution. This element can be transported to meadows through atmospheric deposition (dust), airborne particulates, and water runoff. Subsequently, Sr may enter food chains via bioaccumulation in grazing animals that consume contaminated vegetation (Güler 2007).

Strontium is primarily accumulated in human bone tissue, where it was reported to be associated with enhanced bone formation and the prevention of osteoporosis (Walstra et al. 1999). In addition, strontium demonstrates antioxidant properties that may help to mitigate oxidative stress (and has shown potential in supporting the management of various conditions, including cardiovascular diseases, hypertension, and diabetes) (Basaran and Turk 2024).

#### 3.2.6 Silver (Ag)

The results on the Ag content in the three tested cheese varieties are shown in Table 5. The Ag values significantly differed in the three tested cheese varieties (*p* < 0.01). The Ag contents ranged between 0.38 and 18.96 μg/kg (see Table 5). The average Ag quantity of Telli cheese was higher than that of other samples (*p* < 0.01). Aho and Golot cheese had similar mean Ag values (*p* > 0.05).

Silver (Ag) demonstrates significant antibacterial properties, making it valuable for treating burn wounds, scalds, chronic ulcers, and preventing neonatal conjunctivitis. However, chronic exposure can lead to systemic accumulation of silver particles, resulting in argyria—a characteristic blue‐gray dermal discoloration. Emerging evidence suggests potential genotoxic effects, along with hepatotoxic, nephrotoxic, neurotoxic, and hematological toxicities at elevated exposure levels (Hadrup et al. 2018).

#### 3.2.7 Cadmium (Cd)

The Cd values of the samples did not significantly differ in the three cheeses analyzed in the present study (see Table 5). The Cd content was not determined in 1 out of Golot cheeses and in 1 out of Telli cheese samples. Similarly, Christophoridis et al. (2019) determined that, while Cd was not found in some Greek cheeses, in other cheeses, its content ranged between 0.15 and 1.23 μg/kg. Furthermore, Beikzadeh et al. (2019) found that the Cd contents in Kash (Iranian traditional cheese) samples ranged between 1.541 and 9.940 ppb. Muneam and Abojassim (2023) reported that the highest and mean Cd values of traditional cheese collected from Turkey were 0.375 and 0.167 μg/kg, whereas three of the samples did not have Cd content. Finally, Öksüztepe et al. (2013) found that all Çökelek cheese and Kurut samples did not contain Cd.

Cheeses may become contaminated with cadmium (Cd) during processing, primarily due to contact with processing equipment and exposure to environmental sources (Christophoridis et al. 2019). Exposure to cadmium was previously linked to a range of adverse health effects, including nephrotoxicity, osteoporosis, neurotoxicity, carcinogenic and genotoxic potential, teratogenicity, as well as endocrine and reproductive system disruptions (EFSA 2009). Cadmium primarily exerts its toxic effects on the kidneys, but is also known to contribute to bone demineralization. Furthermore, statistical evidence linked cadmium exposure to an elevated risk of developing cancers in the lung, endometrium, bladder, and breast (EFSA 2012).

#### 3.2.8 Mercury (Hg)

While Hg was not found in all of the tested Aho and Golot cheese samples, it was found in 4 out of 10 Telli cheese samples (see Table 5). The type of cheese had a significant effect on the Hg values (*p* < 0.01) (Table 5). The highest and mean Hg contents of Telli cheese were 468.71 and 154.55 μg/kg, respectively. Similar results were reported by Rocha et al. (2023) and by Dağcilar and Gezer (2021), who found that cheeses did not contain Hg according to the researchers. Furthermore, Christophoridis et al. (2019) reported that Hg was detected in some cheeses in Greece. Conversely, the Hg amounts of other samples were between 0.340 and 1.46 μg/kg.

The presence of mercury (Hg) in dairy products may result from the ingestion of contaminated food and water by dairy animals, as well as from exposure to mercury‐based fungicides and pesticides used in agricultural practices (Christophoridis et al. 2019; Rocha et al. 2023). The kidneys are the primary target organ for the toxic effects of inorganic mercury. However, other systems, including the liver, nervous system, immune system, as well as the reproductive and developmental systems, may also be adversely affected by Hg (Alexander et al. 2008).

#### 3.2.9 Lead (Pb)

The Pb amounts did not significantly differ in the three cheese varieties analyzed in the present study (see Table 5). The Golot cheese samples had the lowest (10.58 μg/kg) and highest (1788.75 μg/kg) Pb contents. The average Pb values ranged between 48.08 mg/kg and 241.87 μg/kg. Kalaycı et al. (2023) found that the mean Ld amount of Çökelek cheese was 0.140 mg/kg. In another relevant study, Rocha et al. (2023) determined that the Pb amount of Brazilian cheese samples ranged between 5.78 and 81.50 μg/kg. Furthermore, the Pb contents of some traditional Turkish cheeses ranged between 0.31 and 1.2 μg/kg (Mendil 2006). These results were lower than those observed in the present study.

Lead (Pb) contamination in cheese may originate from multiple sources, including environmental factors such as water, air, and soil pollution. Furthermore, improper handling during milk transportation or production, particularly through contaminated equipment, can further contribute to Pb exposure (Christophoridis et al. 2019; Dağcilar and Gezer 2021). Pb exposure can lead to numerous health issues, affecting multiple systems such as the nervous, cardiovascular, and immune systems, as well as organs such as the bones, lungs, kidneys, and liver; finally, Pb can impair reproductive function (Basaran and Turk 2024).

#### 3.2.10 Crome (Cr)

As can be seen in Table 5, the Cr content did not significantly differ across the cheese samples (*p* > 0.05). The lowest and highest Cr values were determined in Golot cheese as 17.36 and 341.95 μg/kg, respectively. The lowest mean Cr content was 67.90 μg/kg in Aho cheese, while the highest was 85.43 μg/kg in Telli cheese. In several previous studies, the Cr contents were determined as 0.43–0.48 mg/kg in mozzarella cheese (Capcarova et al. 2024), 0.105 mg/kg in traditional Turkey cheeses (Muneam and Abojassim 2023), and 20.80–146.91 μg/kg in artisanal Brazilian cheeses.

Chromium (Cr), a key component of the glucose tolerance factor, enhances insulin activity and facilitates blood glucose regulation. In addition, Cr plays a crucial role in carbohydrate and lipid metabolism, with deficiency states potentially contributing to metabolic disturbances and weight‐related pathologies (Basaran and Turk 2024). However, available evidence on the potential toxicity or harmful effects of Cr at levels beyond the permissible limit remains scarce (Al Sidawi et al. 2021). The potential chromium contamination in dairy products may arise during milk processing due to contact with stainless steel equipment, which typically contains 10%–30% chromium (Christophoridis et al. 2019).

### 3.3 Health Risk Assessment

The EDI and reference dose (RfD) values for analyzed heavy metals are summarized in Table 6. The results of our comparative analysis revealed that Golot cheese exhibited the lowest mean EDI value for arsenic (As) at 0.002 μg/kg body weight/day, while Aho cheese showed the highest exposure level for strontium (Sr) at 5.66 μg/kg body weight/day. Further research on additives or processing aids, as demonstrated in thermally treated oils (Zhang et al. 2024), may offer important insights into reducing contaminant formation in traditional cheeses.

Quantifiable nickel (Ni) EDI values were obtained for 80% (8/10) of Aho, 70% (7/10) of Golot, and 70% (7/10) of Telli cheese samples. Analytical limitations precluded arsenic (As) EDI determination in 10% (1/10) of Aho, 40% (4/10) of Golot, and 50% (5/10) of Telli samples. Of note, cadmium (Cd) exposure assessment was not feasible for any Aho cheese specimens, with single exclusions occurring in both Golot and Telli varieties (10% each). Mercury (Hg) EDI values were successfully calculated for all Aho and Golot samples; however, only 60% (6/10) of Telli specimens yielded measurable concentrations (Table 6).

The target hazard quotient (THQ) serves as a critical indicator for evaluating potential health risks associated with dietary exposure to heavy metal contamination (Meshref et al. 2014). Quantitative analysis revealed that most cheese samples exhibited THQ values below the safety threshold of 1.0, indicating negligible health concerns for regular consumption. However, the following two exceptions were identified:

A single Golot cheese sample exceeded the threshold for lead (Pb) contamination.

One Telli cheese sample demonstrated elevated mercury (Hg) levels.

Maximum mean THQ values varied significantly among cheese varieties:

Aho cheese: 0.087 μg/kg/day (Pb).

Golot cheese: 0.371 μg/kg/day (Pb).

Telli cheese: 0.395 μg/kg/day (Hg) (Table 7).

**TABLE 7:** THQ and HI values of heavy metal content of cheese samples (μg/kg per day).

Parameters		Aho cheese (*n* = 10)	Golot cheese (*n* = 10)	Telli cheese (*n* = 10)
Ni	Min	—	—	—
	Max	0.011	0.141	0.026
	Mean	0.006 ± 0.007	0.058 ± 0.072	0.016 ± 0.014
	95% CI	0.000–0.065	0.000–0.237	0.000–0.050
Cu	Min	0.006	0.004	0.005
	Max	0.013	0.161	0.015
	Mean	0.010 ± 0.002	0.025 ± 0.048	0.009 ± 0.003
	95% CI	0.009–0.011	0.009–0.059	0.006–0.011
Co	Min	0.006	0.003	0.006
	Max	0.014	0.020	0.035
	Mean	0.010 ± 0.003	0.009 ± 0.005	0.016 ± 0.013
	95% CI	0.008–0.012	0.005–0.012	0.007–0.025
As	Min	—	—	—
	Max	0.023	0.009	0.016
	Mean	0.009 ± 0.007	0.006 ± 0.003	0.012 ± 0.002
	95% CI	0.004–0.014	0.004–0.009	0.009–0.015
Sr	Min	0.007	0.003	0.005
	Max	0.012	0.014	0.011
	Mean	0.009 ± 0.002	0.007 ± 0.004	0.007 ± 0.002
	95% CI	0.008–0.011	0.005–0.010	0.006–0.009
Ag	Min	0.000	0.000	0.001
	Max	0.004	0.001	0.003
	Mean	0.001 ± 0.001	0.001 ± 0.000	0.002 ± 0.001
	95% CI	0.000–0.002	0.000	0.000–0.002
Cd	Min	0.004	—	—
	Max	0.054	0.082	0.075
	Mean	0.016 ± 0.017	0.027 ± 0.026	0.025 ± 0.026
	95% CI	0.004–0.028	0.008–0.047	0.005–0.0045
Hg	Min	—	—	0.067
	Max	—	—	1.199
	Mean	—	—	0.395 ± 0.538
	95% CI	—	—	0.013–0.297
Pb	Min	0.045	0.016	0.025
	Max	0.166	2.744	0.166
	Mean	0.087 ± 0.036	0.371 ± 0.839	0.074 ± 0.040
	95% CI	0.061–0.113	0.000–0.971	0.045–0.102
Cr	Min	0.006	0.004	0.008
	Max	0.077	0.087	0.070
	Mean	0.017 ± 0.021	0.020 ± 0.024	0.022 ± 0.019
	95% CI	0.002–0.033	0.002–0.037	0.009–0.035
HI	Min	0.097	0.052	0.059
	Max	0.296	2.875	1.317
	Mean	0.160 ± 0.063	0.479 ± 0.864	0.321 ± 0.369
	95% CI	0.114–0.205	0.00–1.097	0.057–0.585

Abbreviations: HI: hazard index; THQ: target hazard quotient.

**FIGURE 2:**
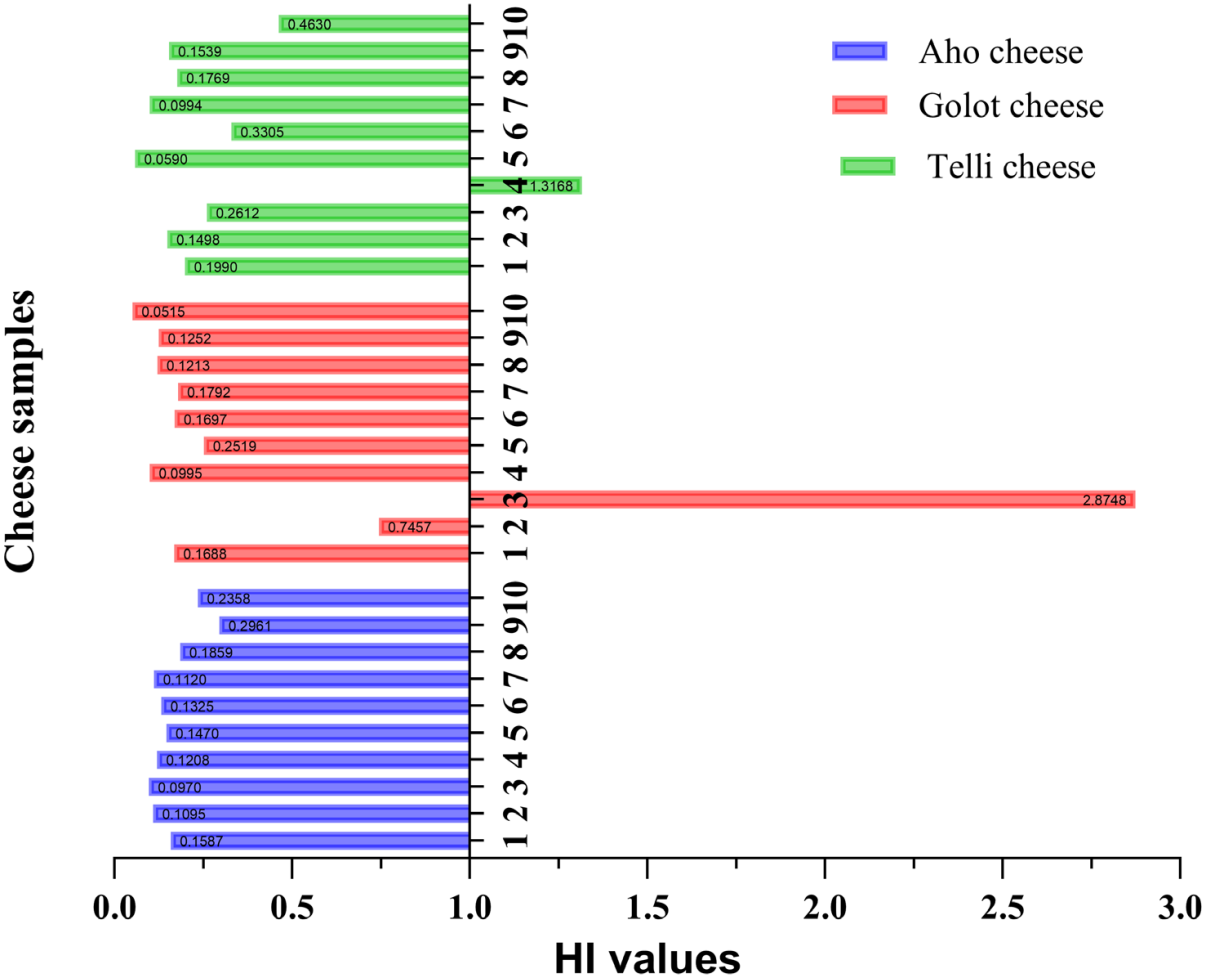
HI values of traditional cheese samples.

Furthermore, the Hazard Index (HI) analysis revealed that the mean values for all cheese types remained below the safety threshold of 1 (see Table 7 and Figure 2), indicating generally negligible health risks from cumulative heavy metal exposure. However, individual sample analysis showed notable variations:

Aho cheese: HI range = 0.097–0.296.

Golot cheese: HI range = 0.052–2.875, with one outlier (2.875) exceeding the threshold.

Telli cheese: HI range = 0.059–1.317, with one sample (1.317) above the safety limit.

According to established risk classification (Basaran 2022; Basaran and Turk 2024):

HI < 1: No significant health concern.

HI 1–5: Low‐level health concern requiring monitoring.

HI 10–100: Substantial health risk demanding intervention.

While 98% of samples (*n* = 28/30) demonstrated acceptable safety levels (HI < 1), two samples (6.7%) fell within the low‐level concern category (HI 1–5). These findings suggest that regular consumption of most tested cheeses poses minimal health risks to consumers, thus warranting more quality control attention. Similarly to advanced glycation end‐products (AGEs) in processed foods, cumulative exposure to heavy metals in traditional cheeses warrants systematic monitoring, as even low‐level contaminants may pose long‐term health risks (Zhang et al. 2024).

## 4 Conclusion

This study provides a holistic evaluation of mineral content and heavy metal contamination in three traditional Turkish cheeses (Aho, Golot, and Telli). These cheeses are rich in essential minerals like Na, Ca, K, and Mg that are beneficial elements for human health, whereas the cheese samples contain heavy metal contamination, including Ni, Cu, Co, As, Sr, Ag, Cd, Hg, Pb, and Cr. Heavy metal analysis identified high concentrations of lead and Hg in some samples, although health risk assessments confirmed that most (93.3%) of the cheese samples did not pose serious risks to consumers. Two outliers with elevated hazard index (HI) values (1–5) warrant attention, suggesting targeted quality control measures for specific production batches.

While the three tested cheeses meaningfully contribute to dietary mineral intake, particularly sodium and calcium, it remains critical to monitor heavy metal contamination in these cheeses, particularly in regions with environmental pollution. To this end, necessary measures should include introducing stricter sanitation protocols during production and storage, establishing routine heavy metal surveillance for high‐risk elements like Pb and Hg, and educating consumers on moderate intake to balance nutritional benefits with potential risks. The producers should also consider the quality of raw milk used, hygienic rules, equipment used in the production, and storage conditions.

By addressing these challenges, traditional Turkish cheeses can continue to be a safe, nutritious, and culturally significant food product for local and global markets. It could also suggest areas for future research, particularly regarding the microbiological safety and nutritional properties of traditional cheeses.

## Author Contributions

**Seyit Erol:** data curation (equal), formal analysis (equal), investigation (equal). **Bayram Ürkek:** data curation (equal), formal analysis (equal), investigation (equal), methodology (equal), project administration (equal), software (equal), supervision (equal), writing – original draft (equal), writing – review and editing (equal).

## Conflicts of Interest

The authors declare no conflicts of interest.

## Data Availability

The datasets generated during and/or analyzed during the current study are available from the corresponding author on reasonable request.
